# Innovations and development of sustainable personal protective equipment: a path to a greener future

**DOI:** 10.1186/s40068-024-00350-x

**Published:** 2024-06-18

**Authors:** Linxiang Lyu, Monisha Bagchi, Nektaria Markoglou, Chunjiang An

**Affiliations:** 1 Department of Building, Civil and Environmental Engineering, Concordia University, Montreal, QC H3G 1M8 Canada; 2 Meltech Innovation Canada Inc., Medicom Group, Pointe-Claire, Montreal, QC H9P 2Z2 Canada

**Keywords:** Personal protective equipment (PPE), Sustainability, Recycling and upcycling, Waste management

## Abstract

The unprecedented surge in the demand for personal protective equipment (PPE) worldwide during the covid pandemic resulted in a significant increase in PPE consumption and subsequent waste generation. Despite the importance of PPE, its widespread usage and disposal have sparked worries about the environmental impact and its long-term sustainability. The increasing awareness of environmental challenges, resource scarcity, and the urgent need to mitigate climate change necessitates a paradigm shift in the product design, manufacturing process, and waste management of PPE. To address these challenges and have a sustainable PPE future, the development of degradable polymers and natural fibers offers a promising alternative to traditional plastics. Additionally, recycling and upcycling methods can convert the waste into valuable alternate products or energy sources, thereby reducing their environmental impact. Better waste management systems, comprehensive policy frameworks, and international collaborations are essential for the effective PPE waste management and the promotion of sustainable practices. Despite the challenges, collaborative efforts across governments, manufacturers, research institutions, and waste management authorities are crucial for transitioning to a more sustainable PPE industry and a circular economy, ultimately benefiting both the environment and society.

## PPE consumption and waste generation

Over the past few years, the global demand of personal protective equipment (PPE) such as gloves, face masks, eyewear, face shields, has seen a significant increase due to the COVID-19 pandemic worldwide (Aragaw and Mekonnen 2021; Khan et al. 2023). Prior to COVID-19, the monthly demand for PPE was estimated at 89 million medical masks, 76 million gloves, and 1.6 million goggles. However, in response to COVID-19, estimated 129 billion masks and 65 billion gloves were consumed globally each month (Zeng et al. 2024). Furthermore, it is anticipated that the supply of medical plastic would increase at a 20% annual rate between 2020 and 2025, reaching approximately 20.9 million tons of medical plastic by 2025 (Zeng et al. 2024). According to Ivanović et al. (2023), gloves are the most commonly used PPE in hospitals, followed by body protection products and masks. Although gloves drive the PPE consumption (quantity), yet they only account for about one-third of the total mass of waste while the body protection products account for maximum mass of waste because of their heavier grammage. Typically, a variety of petroleum-based materials such as high-density polyethylene (HDPE), low-density polyethylene (LDPE), polyvinyl chloride (PVC), polypropylene (PP), and polyester are utilized in the manufacturing of PPE products to meet various safety standards and regulations to protect the end users from specific hazards while also providing durability and comfort. For example, face masks are predominantly made from polypropylene (PP), polystyrene (PS), polycarbonate (PC), polyethylene (PE), or polyester (PES) (Lyu et al. 2024). Disposable gloves, composed of latex, nitrile, and vinyl folic, account for almost 94.6% of all kinds of gloves (Wang et al. 2022). Eye protection such as goggles, safety glasses, or face shields, which are primarily made of polycarbonate (Zeng et al. 2024).

Massive consumption and improper disposal of single use PPE can result to considerable generation of plastic waste, with plastics accounting for more than half of their weight (Singh et al. 2020). The primary methods for PPE waste disposal, landfilling and incineration, exert considerable burden on the PPE waste management system (Lyu et al. 2023b). Furthermore, certain amount of PPE waste is discarded directly into the environment rather than being disposed in landfills or incineration facilities. These PPE wastes are usually made of non-biodegradable materials, such as polypropylene and polyethylene, which means they degrade slowly and can remain in the environment for long periods of time. When these plastic PPE waste end up in the environment, they degrade through a variety of processes, such as physical degradation, photodegradation, and biodegradation. This can result in the release of micro/nano plastics, which are harmful to many biota, compartments, and biological systems (Kiran et al. 2022). Additionally, numerous additives such as plasticizers, synthetic antioxidants, and metal elements, are being added during the manufacturing process, which when released into the environment, may be harmful and have long term effect on the environment (Lyu et al. 2024; Zhang et al. 2024).

Despite the importance of PPE, its widespread usage and disposal have sparked worries about their environmental impact and long-term sustainability. Traditional PPE materials, such as plastics and synthetic fibers, are frequently linked to pollution, resource depletion, and landfill accumulation. Furthermore, the production, delivery, and disposal of PPE items emit considerable amounts of greenhouse gases, compounding the environmental issues we confront (Patrício Silva et al. 2021).

## Application of new and green materials in PPE production

In terms of the long-term management, it is necessary to explore various options to mitigate the environment threat posed by these PPE plastic waste. One of the promising and paramount ways is to develop sustainable options for masks and packaging materials. Biodegradable polymers, which include both natural materials like chitosan, alginate, collagen, and gelatin, and synthetic materials such as polyvinyl alcohol (PVA), polyethylene oxide (PEO), polycaprolactone (PCL), and polylactic acid (PLA), can break down through enzymatic and hydrolytic processes (Lyu et al. 2023a). Several innovative materials have emerged as potential alternatives to conventional plastics and synthetics. For example, graphene microfibre fabric reinforced masks that can be disinfected and reused have been developed due to graphene’s natural antistatic and waterproof properties (De Luca et al. 2023). Natural fibers, such as bamboo and cellulose, can provide a natural, biodegradable, and comfortable material for masks and protective clothing. However, despite the promise of these materials, their cost, availability, and performance need to be considered for their application in PPE production, and further research and development are needed to optimize their use. As sustainable materials become more cost-competitive and their supply chains mature, they are likely to play an increasingly important role in reducing the environmental impact of plastic.

## Recycling and upcycling of PPE waste

Given the global reliance on single-use plastics in addition to the efforts of use green materials, practical and relatively easier alternatives would be to recycle or repurpose PPE waste to alleviate the issue of petroleum-based plastic crisis. Implementing efficient and practical disinfection methods, such as ultraviolet light, ozone gas, and microwave, could enhance the recycling of PPE. That could simultaneously mitigate the existing worldwide pressure on production and reduce the strain on plastic waste collection systems (Patrício Silva et al. 2021). Upcycling methods not only recycle PPE components but also repurpose components of PPE for alternative applications and converts it to products of higher value; potential reuse techniques include thermochemical, mechanical, and chemical processes (Li et al. 2023a; Lyu et al. 2024). For example, plastics from PPE can be recycled into other plastic products, textiles can be repurposed as insulation or new fabrics. Physically, certain PPE materials can be combined into composite materials with improved mechanical qualities, such as concrete for construction and roofing and door panels for homes. Additionally, waste-to-energy chemical technologies, such as pyrolysis, can also offer a sustainable energy source while managing waste. These innovative approaches not only extend the lifecycle of PPE materials but also contribute to the development of more environmentally friendly waste management solutions. To enhance these efforts, robust waste pre-treatment techniques and advancements in recycling and upcycling technologies are required.

## Improved PPE waste management practices

Effective waste management is essential for addressing the challenges posed by PPE waste and promoting the transition towards a more sustainable future (Roberts et al. 2022). Initially, establishing a universally applicable PPE evaluation system is essential to properly monitor and manage PPE waste to reduce environmental challenges, and prepare for any future crises. A comprehensive closed-loop collection system must be established to ensure that discarded PPE are properly gathered and directed towards appropriate recycling or upcycling procedures. Some international firms, such as Loop Industries (US) and Ioniqa Technologies (The Netherlands), are exploring chemical upcycling commercially (Li et al. 2023b). This requires the establishment of approved drop-off places as well as disposal procedures. Public awareness and education efforts are also necessary to educate individuals and businesses on the importance of proper PPE waste disposal. In addition to these strategies, innovation in recycling and upcycling technologies is necessary to overcome existing challenges in processing PPE wastes. These efforts together can significantly enhance waste management, mitigate the environmental impact of PPE waste, and contribute to a more sustainable future.

## Policy and regulation frameworks

In the realm of PPE waste management, policies and regulations play a pivotal role in developing sustainable practices and minimizing their environmental impacts. Regulatory frameworks can be established to ensure proper disposal, recycling, and upcycling of PPE waste, together with clear guidelines for waste segregation, collection, and processing. For example, global standards, such as WHO guidelines and ASTM, can be developed with the focus on the safe disposal and environmental sustainability of PPE waste. Eco-friendly claims involve using recyclable materials and green production methods to minimize PPE waste’s environmental impact. The standard called ‘The Recycled Claim Standard (RCS)’ can ensure that sustainable PPE uses recycled materials with less environmental impact. Additionally, extended producer responsibility (EPR) rules can be introduced to motivate manufacturers accountable for their products’ lifecycles and promote take-back initiatives. Furthermore, incentives such as tax breaks or subsidies can be coupled to motivate businesses to adopt environmentally friendly materials or invest in waste reduction technologies. International cooperation is essential to standardize existing standards and address transboundary concerns, and dedicated funding for research and development can drive advancements in sustainable PPE materials and waste management procedures. Governments, manufacturers, waste management companies, research institutions, and other stakeholders can work together to develop effective waste management solutions, sharing of resources, and best practices. For example, according to the report of National League of Cities, 17 municipalities in the United States, including Miami and Millinocket, have adopted green purchasing standards for Environmentally Preferential Procurement (EPP), which can actively support ‘green’ products that contain reused, recycled, or composted materials, thereby promoting circularity (National League of Cities 2021). These policies should also be clear, enforceable, and adaptable to ensure their ongoing effectiveness and relevance over time.

## Conclusions and perspectives

**Fig. 1:**
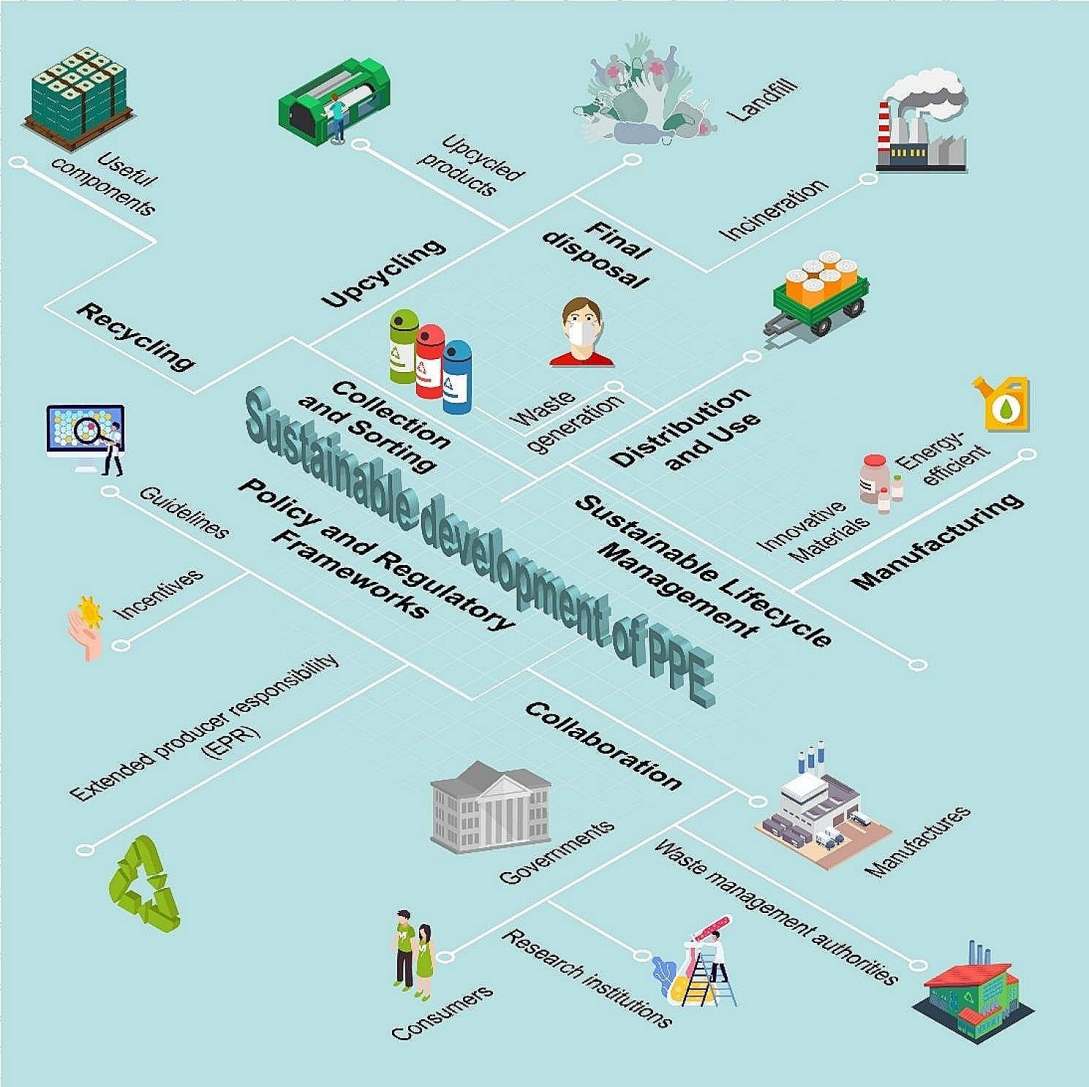
Schematic diagram about the development of sustainable PPE

The escalating global consumption of personal protective equipment (PPE) has led to increased manufacturing and end-user awareness regarding the environmental impact and need of sustainability of these products. The entire lifecycle of PPE, from the production to transportation and its final disposal are areas for generating environmental impact improvements. Addressing the sustainability of PPE encompasses a multifaceted approach, including the application of green materials, the development of PPE recycling and upcycling techniques, the implementation of improved waste management strategies, and the establishment of robust policy frameworks and regulatory mechanisms (Fig. 1). Transitioning to sustainable PPE products and processes mitigates environmental impacts and offers societal benefits. Despite the compelling rationale for sustainability, numerous challenges exist with which efforts and collaboration across sectors and stakeholders, including governments, manufacturers, waste management authorities, research institutions, and consumers, are contributing to the global effort to achieve a more sustainable PPE and a robust circular economy.

## Data Availability

No datasets were generated or analysed during the current study.
